# Effects of hyperbaric oxygen therapy on autistic behaviors and *GRIN2B* gene expression in valproic acid-exposed rats

**DOI:** 10.3389/fnins.2024.1385189

**Published:** 2024-03-18

**Authors:** Yalda Mohtaj Khorassani, Ali Moghimi, Mohammad Reza Khakzad, Masoud Fereidoni, Delaram Hassani, Javad Torbati Gah

**Affiliations:** ^1^Rayan Research Center for Neuroscience & Behavior, Department of Biology, Faculty of Science, Ferdowsi University of Mashhad, Mashhad, Iran; ^2^Innovative Medical Research Center and Department of Immunology, Mashhad Medical Branch, Islamic Azad University, Mashhad, Iran; ^3^Department of Cellular and Molecular Biology, Faculty of Advanced Science and Technology, Tehran Medical Sciences, Islamic Azad University, Tehran, Iran; ^4^Department of Biology, Faculty of Science, Mashhad Islamic Azad University, Mashhad, Iran

**Keywords:** autism, hyperbaric oxygen therapy, *GRIN2B* gene, valproic acid, social interaction

## Abstract

**Introduction:**

Autism is a complex neurodevelopmental condition characterized by deficits in social interaction, communication, and restricted repetitive behaviors. Hyperbaric oxygen therapy (HBOT) has emerged as a potential treatment for autism, although its effects on behavior and gene expression are not well understood. The *GRIN2B* gene, known for its involvement in encoding a glutamate receptor subunit crucial for neuron communication and associated with autism, was a focus of this study.

**Methods:**

Using a rat model induced by prenatal exposure to valproic acid, we examined the impact of HBOT on autism-like behaviors and *GRIN2B* gene expression. Male Wistar rats were categorized into four groups: control, VPA (valproic acid-exposed), VPA+HBOT [2 atmosphere absolute (ATA)], and VPA+HBOT (2.5 ATA). The rats underwent several behavioral tests to assess social behavior, anxiety, stereotype and exploratory behaviors, and learning. Following the behavioral tests, the HBOT groups received 15 sessions of HBOT at pressures of 2 and 2.5 (ATA), and their behaviors were re-evaluated. Subsequently, real-time PCR was employed to measure *GRIN2B* gene expression in the frontal lobe.

**Results:**

Our results indicated that HBOT significantly increased social interaction and exploratory behaviors in VPA-exposed rats, alongside elevated *GRIN2B* gene expression in their frontal lobe.

**Discussion:**

Our findings imply that HBOT might have a potential role in ameliorating autism-related behaviors in the VPA rat model of autism through potential modulation of *GRIN2B* gene expression. However, additional research is essential to fully comprehend the underlying mechanisms and refine the HBOT protocol for optimizing its effectiveness in improving autism-related symptoms.

## Introduction

Autism is a neurodevelopmental condition in which the development of the brain is affected and it’s characterized by difficulties with social communication and interaction as well as restricted and repetitive interests and behaviors (American Psychiatric Association, 2013). While memory difficulty is not a diagnostic criterion for autism, studies indicate learning and memory impairments in individuals with autism (Goh and Peterson, 2012; Wang et al., 2017). According to the World Health Organization, approximately 1 in 100 children worldwide is diagnosed with autism (Autism, 2023). Autism manifests on a spectrum, varying in symptom severity, associated traits, and accompanying conditions. As a result, there is no one-size-fits-all approach to autism therapies (Malik-Soni et al., 2021).

Glutamate is a major excitatory neurotransmitter and has a key role in neural plasticity as well as cognitive processes including memory and learning (Yoo et al., 2012). Altered glutamatergic pathways are associated with autism (Nisar et al., 2022), potentially contributing to behavioral and cognitive deficits observed in the spectrum.

Evidence suggests autism affects both metabotropic and ionotropic glutamate receptors, implicating glutamate receptor dysregulation in autism’s manifestation (Choudhury et al., 2012). N-methyl-d-aspartate (NMDA) receptors (NMDAR) are one of the several glutamate receptor subtypes that may disclose more physiological mechanisms and even offer promising probable therapeutic options for autism. NMDA receptors are neurotransmitter-gated ion channels and NMDAR-mediated signaling has a role in proper development, plasticity, learning, memory, and high cognitive functions (Burnashev and Szepetowski, 2015). A study on valproic acid (VPA)-exposed mouse showed that activation of NMDAR regulated sociability (Burket et al., 2015).

It is well known that many of the physiological and pharmacological characteristics of NMDA receptors are influenced by the identification of their specific subunits (Cull-Candy and Leszkiewicz, 2004). NMDARs are heterotetrametric structures usually composed of two GluN1 subunits and two GluN2 subunits, comprising four subtypes (GluN2A-D) (Cull-Candy et al., 2001). Mutations in NMDAR subunits and changes in gene expression are implicated in various neurodevelopmental disorders, including autism (Rinaldi et al., 2007; Yoo et al., 2012; Burnashev and Szepetowski, 2015). The *GRIN2B* gene has been shown to have an important role in behavior. A study showed that the variations present in the *GRIN2B* gene have been associated with the decision-making process (Ness et al., 2011). Also, it has been demonstrated that *GRIN2B* plays a critical role in working memory (Pergola et al., 2016). Variations in the *GRIN2B* gene correlate with obsessive-compulsive disorder and its symptom dimensions (Kohlrausch et al., 2016). Genetic findings suggest *GRIN2B* involvement in schizophrenia and bipolar disorder pathophysiology (Martucci et al., 2006).

The frontal cortex, linked to higher cognitive functions and emotional regulation, significantly influences autism-related behaviors (Collins and Koechlin, 2012; Jones and Graff-Radford, 2021; Friedman and Robbins, 2022; Mohapatra and Wagner, 2023). Altered frontal cortex activity and connectivity have been implicated in social interaction deficits, repetitive behaviors, and cognitive impairments observed in individuals with Autism (Hou et al., 2018; Margari et al., 2018; Zhu et al., 2022). Therefore, investigating the relationship between glutamatergic signaling, NMDA receptors, and the prefrontal cortex is important for understanding the neurobiology of autism.

There are anecdotal reports of individuals with autism experiencing symptom improvements through Hyperbaric Oxygen Therapy (HBOT) (Rossignol et al., 2007, 2012; Chungpaibulpatana et al., 2008; Rossigno et al., 2009; Hao et al., 2020). Exploring these reports could guide further investigations. Exploring the reported anecdotal benefits of HBOT in individuals with autism offers an avenue for further investigation, potentially paving the way for a non-invasive treatment option if proven effective through rigorous clinical trials.

HBOT involves inhaling nearly 100% oxygen at pressures exceeding sea level pressure (1 ATA) within a hyperbaric chamber [Undersea and Hyperbaric Medical Society (UHMS), 2019]. So far, HBOT has shown promise in enhancing cognitive function, spatial learning, and memory post-brain injury (Harch et al., 2007; Liu et al., 2013), and exhibits neuroprotective effects (Yang et al., 2023).

Nevertheless, the effects of HBOT on autism are contradictory, and controlled clinical trials are limited (Granpeesheh et al., 2010; Jepson et al., 2011; Sampanthavivat et al., 2012). Thus, the present study aimed to investigate the effect of HBOT at pressures of 2 and 2.5 atmosphere absolute on autistic-like behaviors and *GRIN2B* gene expression in the VPA-induced rat model of autism.

## Materials and methods

### Animals and groups

Male and female Wistar rats, nearly three months old and weighing approximately 200–250 grams, were housed in plastic cages. A total of 11 female and 11 male rats were utilized for the experimental procedures. Each female rat was involved in a single pregnancy only, and none were subjected to a second pregnancy during the study. All animals were kept in standard laboratory conditions at a temperature of 22 ± 2 degrees Celsius, with around 50% humidity, and a 12-h light/dark cycle. Access to food and water was provided *ad libitum*. Mating cages were set up for male and female rats. The appearance of vaginal plugs was considered as the zero-day of pregnancy (Goyeneche et al., 2003). Pregnant rats received a single intraperitoneal injection of 500 mg/kg VPA (Sajaddaru Co., Iran) dissolved in 3.3 mL/kg saline on embryonic day 12.5, except for the control group which did not receive any injections (Favre et al., 2013). Pups were weaned on postnatal day 21 and they were examined for morphological malformation (Saft et al., 2014). Subsequent experiments were conducted on the male offspring. The pups were categorized into four groups:

(1) The intact control group consisted of wildtype rats (no interventions were administered), (2) Sham, VPA-exposed rats, received prenatal VPA but did not undergo any oxygen treatment, (3) HBOT-2 group comprised VPA-exposed rats that received hyperbaric oxygen therapy at 2 ATA and (4) the HBOT-2.5 comprised VPA-exposed rats that received hyperbaric oxygen therapy at 2.5 ATA.

### Ethical consideration

All Experiments were carried out in accordance with the international ethical codes and complied with local institutional regulations at Ferdowsi University of Mashhad (Iran National Committee for Ethics in Biomedical Research). The project obtained ethical approval from the local FUM Ethical Review Committee (IR.UM.REC.1399.117). The study’s reporting follows the ARRIVE guidelines.

### Behavioral evaluation

Four days after separation from their mother, the pups underwent the three-chamber social interaction test, Y-maze, elevated plus maze, and Morris Water Maze (MWM) tests. These evaluations assessed social behaviors, repetitive behaviors, anxiety, and spatial memory, respectively.

All behavioral assessments were conducted on 25-day-old rats, between 9 am and 4 pm. One hour before testing, all animal-containing cages were transferred to the laboratory to allow the animals to habituate to the testing room (Hånell and Marklund, 2014).

### Social interaction test

This test assesses two key aspects of social behavior: “Sociability,” which is defined as the propensity to spend time with another rat rather than with a nonliving object, and “Social Novelty” which is the preference for spending time with an unfamiliar rat over a familiar one (Moy et al., 2004). The apparatus used was a box (100 × 40 × 40 cm) divided by separating walls, allowing free access to each chamber. The side chambers contained two identical wire cages. The test comprised four 10-min stages separated by 5-min intervals. To prevent residual odors from influencing the test, all three chambers and cages were cleaned with 75% ethanol both initially and before each stage. In the first stage (habituation), animals were allowed to freely explore the empty chambers and cages. In the second stage called pre-test, two identical nonliving objects were placed in the cages. The third stage was the Social Stimulus Stage: One wire cage contained an unfamiliar rat (social stimulus), while the opposite cage contained an unfamiliar inanimate object (non-social stimulus). In the last stage (Social Novelty Stage), the non-social object was replaced with a novel unfamiliar rat as the novel social stimulus and the social stimulus were at the same place. During these stages, interactions such as following, touching, grooming, and sniffing any body part were recorded using a video camera. The duration of interaction with each stimulus was manually measured. Animals used as stimuli were randomly chosen, matching the test rats in age, sex, and strain (Rein et al., 2020).

### Elevated plus maze

This test aimed to measure anxiety-like behavior in rats. Rats were placed for 5 min in an apparatus consisting of two open arms and two closed arms (each arm 50 × 10 cm and the height 50 cm). Animals were positioned in the middle of the maze and time spent in each arm was recorded with a video camera connected to a computer using ANY-maze software. The maze was cleaned with 75% ethanol and allowed to dry after each test to eliminate the odors of previously tested *rats*. The total distance traveled and time spent in each arm was recorded (Markram et al., 2008; Hånell and Marklund, 2014).

### Y-maze

The Y-maze can be used to measure short-term memory, exploratory, and stereotyped patterns of behavior (Onaolapo, 2012; Wöhr et al., 2015). Hence, spontaneous alternation was determined using a Y-maze. The maze, consisting of three equal arms (40 × 5 × 30 cm) separated by 120-degree angles, resembles the shape of a Y. Rats were placed individually in the start arm and allowed to explore freely for 5 min (Onaolapo, 2012). The arms were labeled A, B, and C. A video camera connected to a computer running ANY-maze software tracked and recorded the sequence of arm entries and the total distance traveled. An alternation was defined as entry into all three arms consecutively. The percentage of alternation was computed using the formula: [(number of *alternations*) / (total arm entries −2)] × 100. After each trial, the apparatus was cleaned with 75% ethanol and allowed to dry to remove any residual odors from previous rat trials (Onaolapo, 2012; Hånell and Marklund, 2014; Jung et al., 2020).

### Morris water maze (MWM)

The MWM assesses spatial learning and memory capabilities (Vorhees and Williams, 2006). The apparatus consisted of a circular black pool, 150 cm in diameter and 50 cm deep, filled halfway with water maintained at approximately 25 ± 1°C. A platform, matching the pool’s color and measuring 9 cm in diameter and 25 cm in height, was submerged 1 cm below the water’s surface, making it nearly invisible during training sessions. On the first day, the platform was positioned 1 cm above the water’s surface and marked with a red flag for visibility by the animals. The pool was enclosed with curtains to minimize distractions for the animals. Inside the pool, above the water’s surface, four high-contrast spatial cues were placed. The pool was divided into four quadrants by two axes, with directional markers (North, South, East, and West) at the ends of each line. Rats underwent 5 days of spatial training, with each session comprising five 60-s trials and 60-s inter-trial intervals. At the start of each trial, the rat began facing the pool’s wall at one of four positions. If the rat located the platform within 60 s, it remained on the platform for 5 s. However, if the rat did not find the platform, it was gently placed on the platform for 20 s. The escape latency and total distance traveled were automatically recorded using the ANY-maze video tracking software (Vorhees and Williams, 2006; Markram et al., 2008; Bromley-Brits et al., 2011).

### Hyperbaric oxygen treatment (HBOT)

HBOT was administered to 35-day-old rats using a hyperbaric chamber (Irsa Sakht Asia Co., IR). For this purpose, rats were placed in the hyperbaric chamber. After sealing the chamber, the pressure inside the chamber was gradually increased over a 5-min period until it reached the designated pressure levels. One group was pressurized to 2 ATA, while the second group underwent pressurization to 2.5 ATA. Once the chamber pressure attained the desired levels, the rats remained exposed to this pressure condition for 50 min. Throughout this time, a 100% oxygen environment was maintained. The oxygen flow rate was 6 liters per minute. Following the 50-min exposure, the chamber pressure was gradually reduced over a 5-min period to return to atmospheric conditions, totaling the rats’ exposure duration within the chamber to 60 min. After each HBOT session, the rats were returned to their cages. These sessions were conducted once daily for 14 consecutive days (Simsek et al., 2012; Körpınar and Uzun, 2019).

All administrations were conducted between 9 AM and 11 AM to minimize potential effects.

Post-HBOT behavioral tests:

All behavioral tests were repeated after the completion of the HBOT sessions.

### Tissue preparation

For tissue sampling, CO2 was used for euthanizing the animals. After opening the skulls, the whole brain was extracted, and the frontal lobe was immediately excised. The excised frontal lobe samples were placed in microtubes containing RNA later solution (Sinaclon Co., Iran) and stored at −80°C until molecular examinations commenced.

### Real-time PCR

RNA extraction and cDNA synthesis were performed using RNX Plus (Sinnaclon Co, Iran) for total cellular RNA extraction from frontal lobe samples, following the manufacturer’s instructions. Primer sequences for the target gene and *GAPDH* gene (used as a reference gene) were designed and determined (shown in Table 1). The RNA was reverse transcribed into single-stranded cDNA using a cDNA synthesis kit (Pars tous Co, Iran) in accordance with the manufacturer’s protocol.

**Table 1:** Forward and reverse sequences of the *GRIN2B* and *GAPDH* gene.

*GRIN2B*	Forward	CCCTGGCTACCAGGACTTTG
	Reverse	GATGGGGCTTTGGAGCTTCT
*GAPDH*	Forward	CAGCAACTCCCACTCTTCCAC
	Reverse	GTGGTCCAGGGTTTCTTACTC

The real-time PCR procedure was conducted utilizing a Corbett real-time PCR apparatus. The cDNA samples were amplified employing SYBR-Green in a 20 μL reaction mixture comprising 10 μL of 2X TaqMan Universal PCR Master Mix, 1 μL of 20X TaqMan Gene Expression Assay, and 9 μL of cDNA template. The amplification process involved an initial denaturation step at 95°C for 10 min, followed by 40 cycles of denaturation at 95°C for 15 s, and annealing and extension at 60°C for 1 min. Subsequently, the real-time PCR data were analyzed using the comparative Ct method to ascertain the relative expression levels of the target genes, which were then normalized to the expression of the reference gene (Schmittgen and Livak, 2008). To confirm the specificity of the PCR products, a melting curve analysis was performed. Additionally, non-template controls were included in each run to identify any potential contamination or background signal.

### Statistical analysis

The Behavioral tests and Real-time PCR results underwent analysis in R software (R Core Team, 2022). Initially, the data for each test were evaluated for normal distribution using the Shapiro–Wilk test. Subsequently, for the three-chamber social interaction test, exploratory behavior, and spontaneous alternation in the Y-maze test, the Welch *t*-test was used to compare the control group to each treatment group. For the elevated plus maze data, the non-parametric Mann–Whitney test was employed. Then, the paired *t*-test was applied to assess the pre-and post-treatment data for comparison. The MWM data underwent analysis using a repeated measure analysis of variance (ANOVA), adjusted with the Bonferroni correction, followed by Tukey’s *post hoc* test. Gene expression data were analyzed using one-way ANOVA, and Tukey’s *post hoc* test was conducted to determine the specific differences among groups. The repeated measure ANOVA was performed using the nlme package (Pinheiro and Bates, 2000), while *post hoc* tests were conducted using functions from the multcomp package (Hothorn et al., 2008). A significance level of *p*-value ≤0.05 was considered. The statistics reported in the text and figures represent the mean ± SEM.

## Results

### Tail malformation

**Figure 1:**
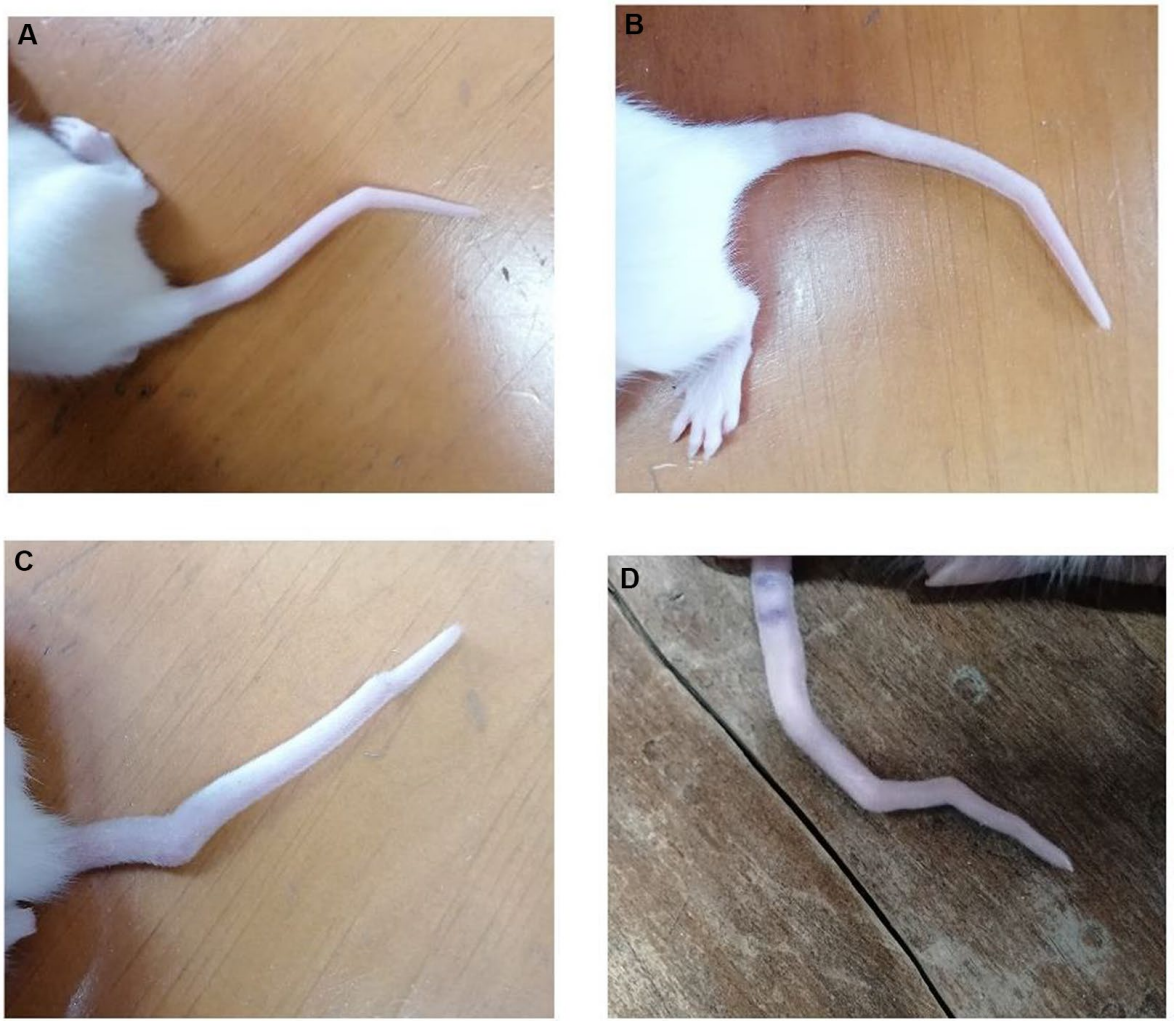
Tail malformation in rats exposed to *in utero* VPA. **(A)** One bent in the tail. **(B,C)** Two bents in the tail. **(D)** Four bents in the tail.

Out of the 36 rats exposed to VPA *in utero*, 28 exhibited tail malformations, indicating that 80% of the VPA-exposed rats showed abnormalities in their tails. Apart from tail malformations, no other body malformations were observed (Figure 1).

The observed tail malformations were classified into two groups: (1) Rats that exhibited a single bend along their tail, and (2) Rats that displayed multiple bends along their tail, often having two bends, with one case showing four bends (Figure 1).

### Behavioral tests

#### Three-chamber social interaction test

##### Social preference

**Figure 2:**
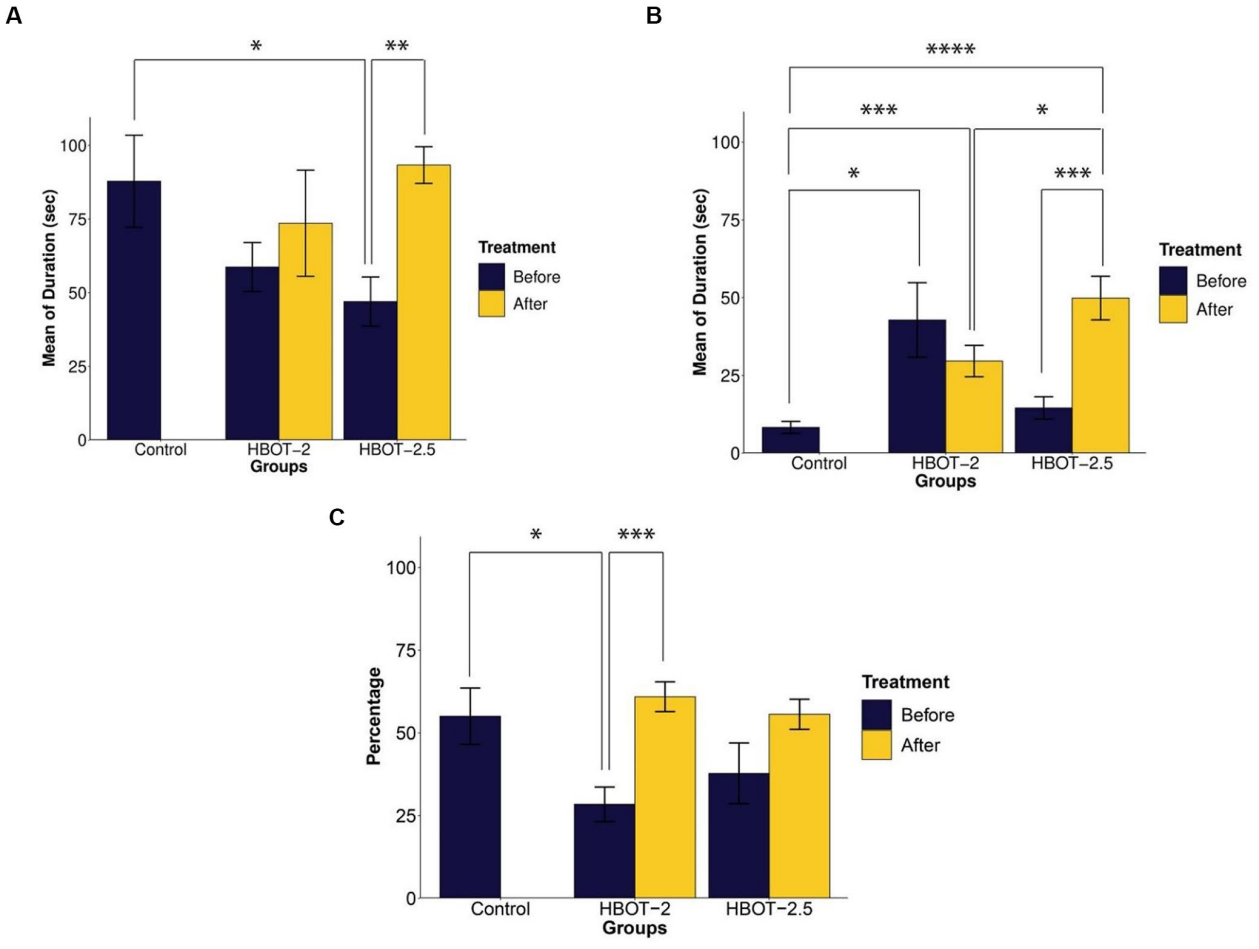
The effects of HBOT on social behavior using the Three-chamber Social Interaction test. **(A)** Duration of interaction with the social stimulus: VPA-exposed rats displayed reduced interaction time, and HBOT notably increased this behavior. **(B)** Interaction time with the non-social stimulus: VPA-exposed rats exhibited more interest in communicating with the non-social stimulus, with varying effects observed post-hyperbaric oxygen therapy across groups. **(C)** Percentage of time spent with the unfamiliar social stimulus: VPA-exposed rats showed decreased interaction time with the unfamiliar stimulus, which was enhanced following HBOT (*p-value ≤ 0.05, **p-value *≤* 0.01, ***p-value *≤ 0.001*, ****p-value ≤ 0.0001). *n*(Control) = 9, *n*(HBOT-2) = 11, *n*(HBOT-2.5) = 12.

Prior to treatment, HBOT-2 rats spent relatively less time with the social stimulus compared to the control group, although this difference was not statistically significant (*t* = 1.6247, df = 12.687, *p*-value = 0.1288). However, significant differences were observed when comparing the control group with HBOT-2.5 (*t* = 2.2817, df = 12.771, *p*-value = 0.04032) (Figure 2A).

In comparison to VPA-exposed rats, control animals spent significantly less time with the non-social stimulus. This difference was statistically significant between the control and VPA-exposed rats of the HBOT groups [control and HBOT-2 (*t* = −2.5722, df = 8.3353, *p*-value = 0.03194), control and HBOT-2.5 (*t* = −1.4812, df = 14.668, *p*-value = 0.1597)] (Figure 2B).

Post-treatment, there was no significant difference observed in the duration of interaction with the social stimulus within the HBOT-2 group (*t* = −0.28618, df = 9, *p*-value = 0.7812). Conversely, the HBOT-2.5 group exhibited a significant increase in time spent with the social stimulus post-treatment (*t* = −3.8013, df = 10, *p*-value = 0.003478) (Figure 2A).

Comparison of the time spent with social stimulus between the control group with the post-treatment data of both HBOT-2 (*t* = 0.59695, df = 17.983, *p*-value = 0.558) and HBOT-2.5 (*t* = −0.32648, df = 10.545, *p*-value = 0.7505) groups did not yield a significant difference (Figure 2A).

After receiving HBOT sessions, HBOT-2 rats spent less time with the non-social stimulus (*t* = 0.77831, df = 8, *p*-value = 0.4588). Conversely, HBOT-2.5 rats exhibited a significant increase in interaction duration with the non-social stimulus post-treatment (*t* = −4.2893, df = 10, *p*-value = 0.001588) (Figure 2B).

Comparatively, VPA-exposed rats interacted significantly more with the non-social stimulus after treatment in comparison to control rats [control and HBOT-2 (*t* = −3.9507, df = 12.784, *p*-value = 0.001711), control and HBOT-2.5 (*t* = −5.7189, df = 12.62, *p*-value = 7.925e-05)] (Figure 2B).

### Social novelty

In the novelty preference test, the percentage of s interaction’animal with the unfamiliar social stimulus has been measured by calculating the interaction time with the unfamiliar stimulus compared to the total interaction time (time interacted with unfamiliar social stimulus/(time interacted with unfamiliar social stimulus + time interacted with familiar social stimulus) × 100).

Before treatment, VPA-exposed rats exhibited impaired preference in interacting with the unfamiliar social stimulus compared to the control group [HBOT-2 and control (*t* = 2.5196, df = 11.854, *p*-value = 0.02714) and HBOT-2.5 and control (*t* = 1.3114, df = 16.765, *p*-value = 0.2074)] (Figure 2C).

Following HBOT administration, interaction with the unfamiliar social stimulus increased in both groups, with statistical significance observed only in HBOT-2 [HBOT-2 before and after (*t* = −4.4773, df = 9, *p*-value = 0.001538)], while not in HBOT-2.5 [HBOT-2.5 before and after (*t* = −2.0733, df = 10, *p*-value = 0.06492)] (Figure 2C).

Comparison of post-treatment data with the control group did not reveal a significant difference [HBOT-2 and control (*t* = −0.58468, df = 10.445, *p*-value = 0.5712), HBOT-2.5 and control (*t* = −0.058536, df = 10.545, *p*-value = 0.9544)] (Figure 2C).

### Elevated plus maze

In this test, the percentage of time spent in the open arms relative to the total test time was calculated to measure anxiety (time spent in the open arms/total test time × 100). Similarly, using the same equation, the percentage of distance traveled in the open arms was also computed.

**Figure 3:**
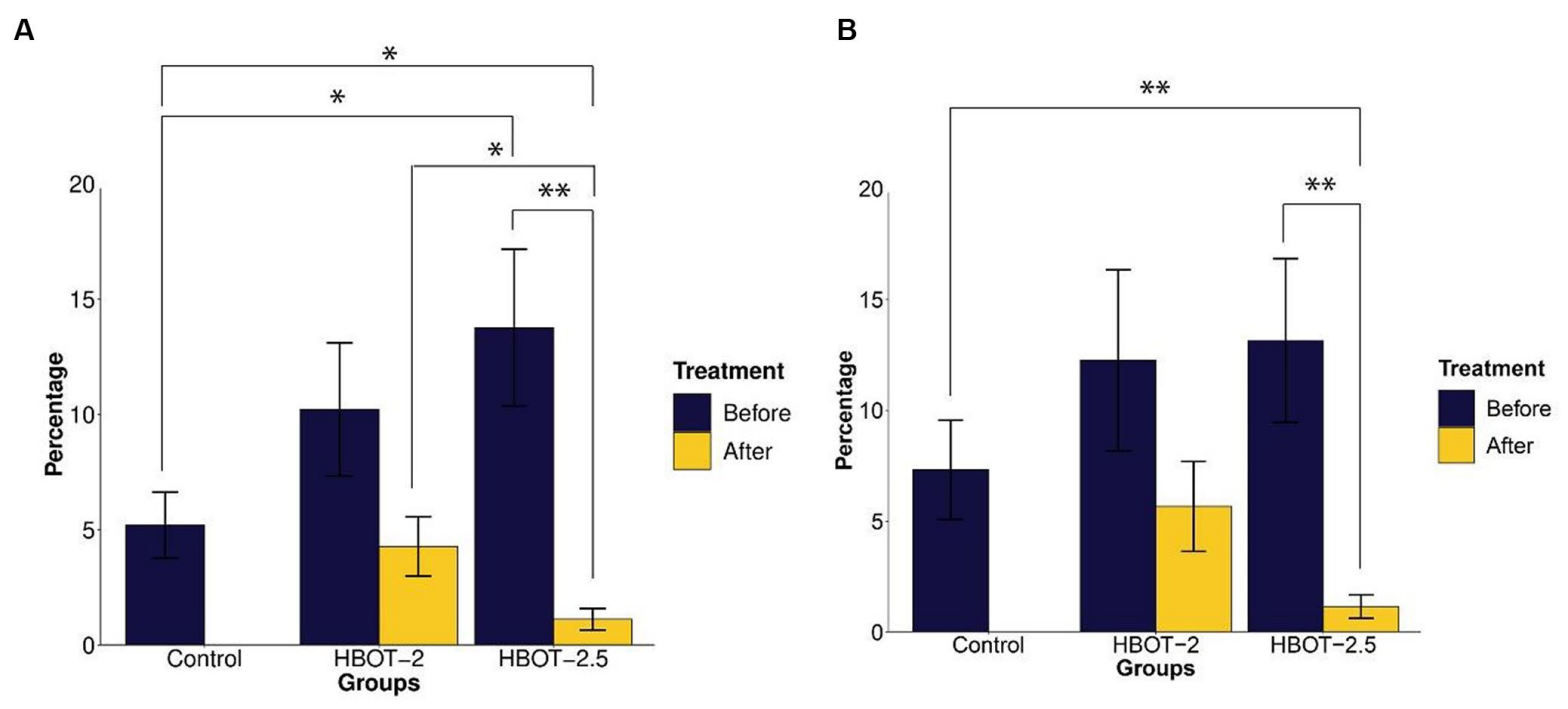
The effects of HBOT on elevated plus maze. **(A)** Percentage of time spent in the open arms: VPA-exposed rats spent more time in the open arms compared to the control group. Following HBOT sessions, both groups showed reduced time spent in the open arms. **(B)** Percentage of total distance traveled in the open arms: VPA-exposed rats traveled a greater distance in the open arms, but this distance decreased after HBOT sessions. (*p-value *≤* 0.05, **p-value ≤ 0.01). *n*(Control) = 9, *n*(HBOT-2) = 11, *n*(HBOT-2.5) = 12.

Compared to control rats, VPA-exposed rats exhibited increased time spent in the open arms. This difference was statistically significant in HBOT-2.5 (*t* = −2.3226, df = 14.61, *p*-value = 0.03509), while not significant in HBOT-2 (*t* = −1.5596, df = 14.427, *p*-value = 0.1405) (Figure 3A).

Post HBOT administration, both groups displayed a decrease in the percentage of time spent in the open arms. This reduction was statistically significant in HBOT-2.5 (*t* = 3.7104, df = 11, *p*-value = 0.003439), but not in HBOT-2 (*t* = 1.7032, df = 10, *p*-value = 0.1194) (Figure 3A).

Comparing control rats with HBOT-2 rats after treatment did not reveal a significant difference (*t* = 0.47785, df = 17.177, *p*-value = 0.6388). However, a significant difference was observed between control rats and HBOT-2.5 rats after HBOT sessions (*t* = 2.7111, df = 9.7132, *p*-value = 0.02243) (Figure 3A).

While VPA-exposed rats showed increased distance traveled in the open arms compared to the control group, this difference was not statistically significant [control and HBOT-2 (*W* = 42.5, *p*-value = 0.6213), control and HBOT-2.5 (*W* = 37, *p*-value = 0.2469)]. Post HBOT sessions, there was a decrease in the percentage of distance traveled in the open arms, notably significant in the HBOT-2.5 group [HBOT-2.5 before and after (V = 75, *p*-value = 0.002441), HBOT-2 before and after (V = 47, *p*-value = 0.2402)]. Furthermore, a significant difference was observed between HBOT-2.5 after receiving HBOT and the control group [HBOT-2.5 and control (*W* = 91.5, *p*-value = 0.006846), HBOT-2 and control (*W* = 59.5, *p*-value = 0.4703)] (Figure 3B).

### Y-maze

#### Exploratory behavior

**Figure 4:**
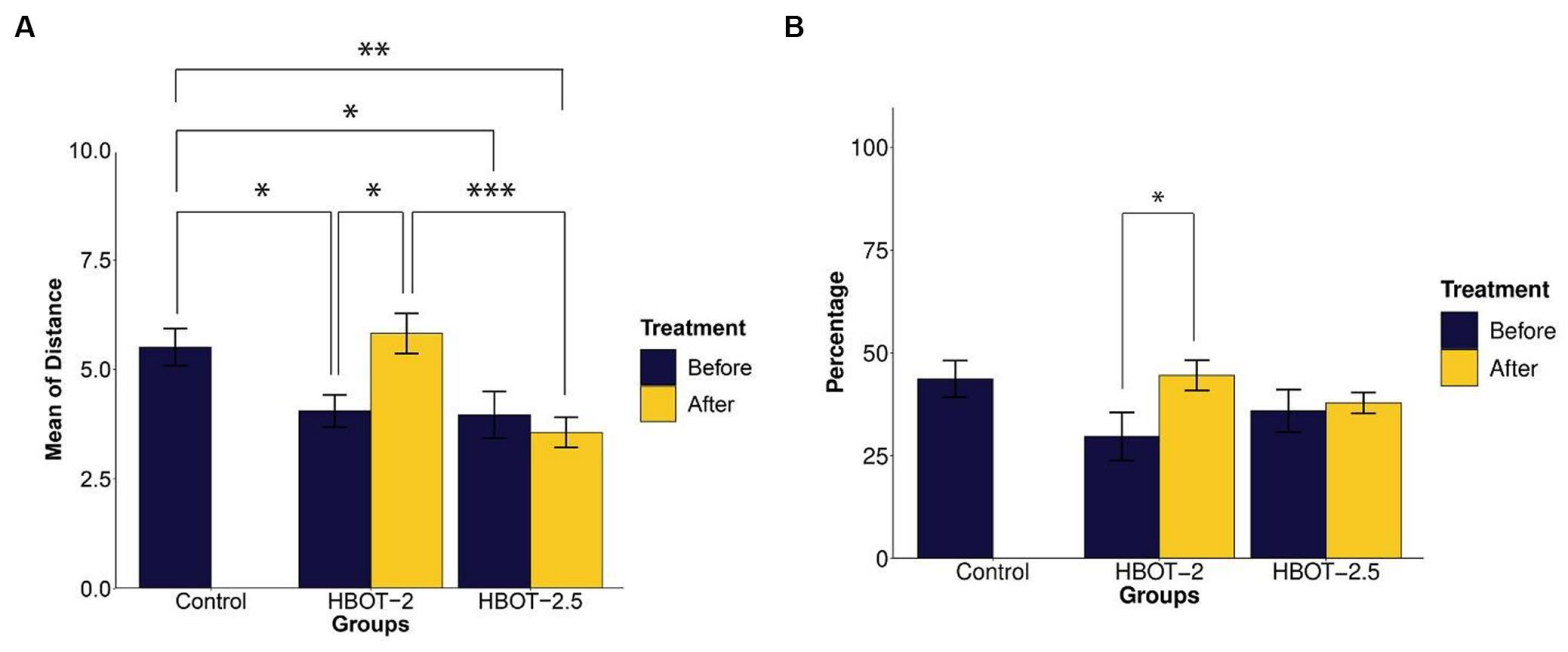
The effects of HBOT on exploratory and spontaneous behavior in Y-maze. **(A)** Average distance traveled by rats: VPA-exposed rats traveled less than the control rats. HBOT at 2 ATA increased exploratory behavior, while 2.5 ATA showed no significant impact. **(B)** Percentage of spontaneous alternation behavior: No significant differences in repetitive behavior were observed between the control and VPA-exposed rats. However, the HBOT-2 group showed a significant increase in SAB percentage, indicating reduced repetitive behavior post-hyperbaric oxygen therapy. (*p-value ≤ 0.05, **p-value ≤ 0.01, ***p-value ≤ 0.001). *n*(Control) = 9, *n*(HBOT-2) = 11, *n*(HBOT-2.5) = 12.

Exploratory behavior was assessed based on the distance traveled by the animals. The data indicated that VPA-exposed animals traveled a significantly shorter distance during the test [HBOT-2 and control (*t* = 2.5095, df = 14.887, *p*-value = 0.02415), HBOT-2.5 and control (*t* = 2.2142, df = 17.951, *p*-value = 0.04)] (Figure 4A).

Post-treatment with hyperbaric oxygen, HBOT-2 rats significantly increased their travel distance compared to before treatment (*t* = −2.4803, df = 10, *p*-value = 0.03253), while HBOT-2.5 rats exhibited a decrease, although not significant (*t* = 0.6981, df = 11, *p*-value = 0.4996) (Figure 4A).

After HBO administration, no significant difference was observed between HBOT-2 and the control group (*t* = −0.48742, df = 16.573, *p*-value = 0.6323). However, a statistically significant difference was found between HBOT-2.5 and the control (*t* = 3.4413, df = 14.449, *p*-value = 0.003814) (Figure 4A).

#### Spontaneous alternation behavior

Spontaneous alternation behavior (SAB) assessed repetitive behavior. As shown in Figure 4B, no statistically significant differences were observed in the percentage of SAB between the control rats and VPA-exposed rats before intervention [control and HBOT-2 (*t* = 1.8551, df = 16.982, *p*-value = 0.08102), control and HBOT-2.5 (*t* = 1.1041, df = 17.667, *p*-value = 0.2844)].

Comparing pre-and post-treatment data indicated a significant increase in SAB in HBOT-2 rats after receiving HBOT (*t* = −2.4169, df = 10, *p*-value = 0.03625). Although there was a slight increase in HBOT-2.5 rats, it was not significant (*t* = −0.28192, df = 11, *p*-value = 0.7832) (Figure 4B).

When comparing control rats with those that received hyperbaric oxygen, no differences were observed [control and HBOT-2 (*t* = −0.14608, df = 14.238, *p*-value = 0.8859), control and HBOT-2.5 (*t* = 1.0794, df = 10.998, *p*-value = 0.3035)] (Figure 4B).

### Morris water maze

**Figure 5:**
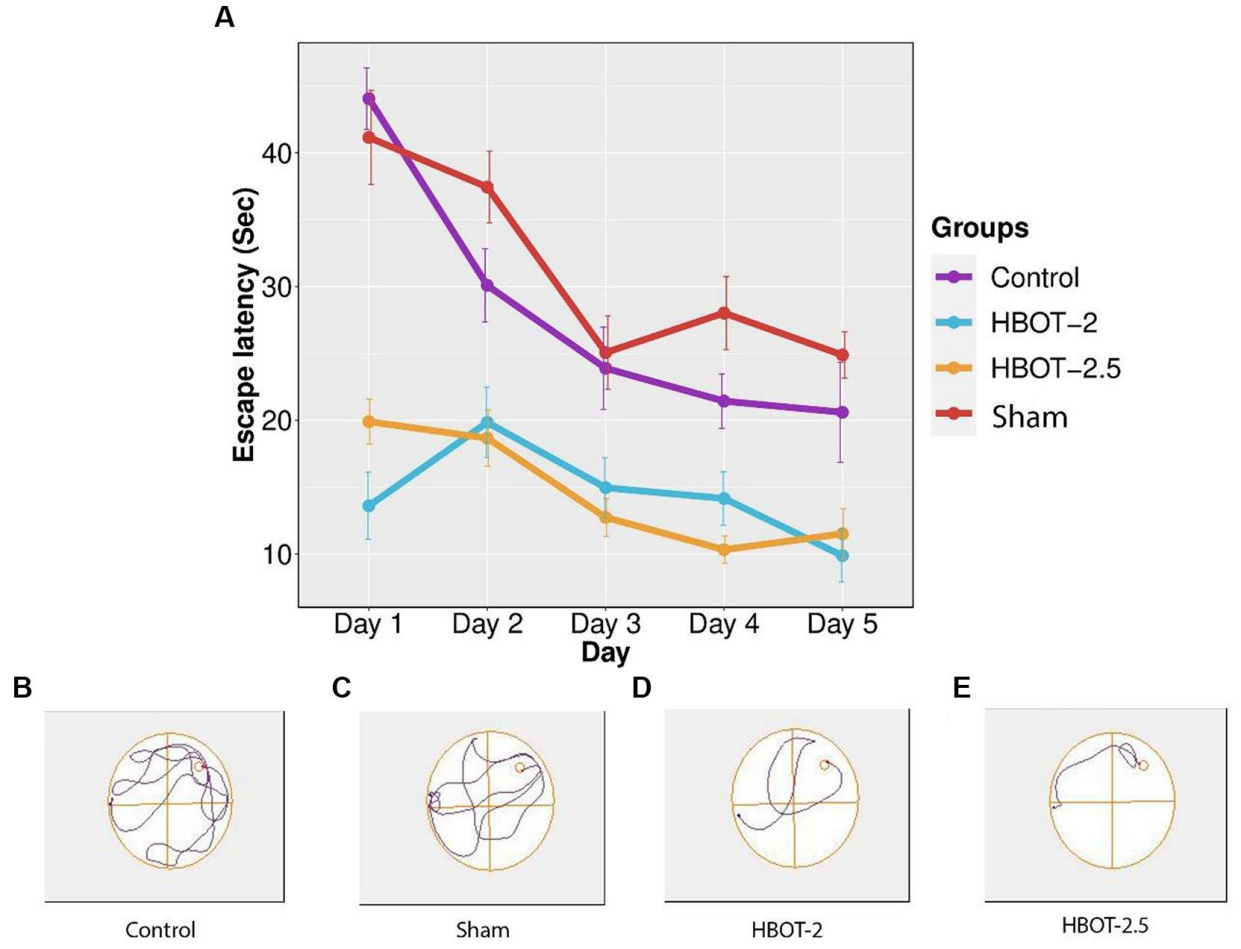
The effects of HBOT on learning using Morris Water Maze. **(A)** Escape latency to reach the platform within 5 days of the test: There was no significant difference in escape latency between the control and sham rats. However, both HBOT-receiving groups showed significantly lower escape latency compared to the control and sham groups. **(B–E)** Track plot of the swimming path. *n*(Control) = 9, *n*(sham) = 12, *n*(HBOT-2) = 11, *n*(HBOT-2.5) = 12.

The maze was used to assess any impairments in learning and memory. Results from the repeated measure ANOVA indicated significant differences between groups (*p*-value <0.0001). As shown in Figure 5, the learning process was observed in the sham rats, yet no statistical differences were found between the control and the sham animals throughout all five days of training (*p*-value = 0.491).

Post-treatment analysis revealed a noteworthy decrease in the time taken by HBOT-2 rats to find the platform after receiving hyperbaric oxygen therapy, compared to the sham and control groups that did not receive HBOT (Sham and HBOT-2: *p*-value <2e-16, Sham and HBOT-2.5: *p*-value <2e-16, Control and HBOT-2: *p*-value = 1.75e-11, Control and HBOT-2.5: *p*-value = 1.24e-11) (Figure 5A).

However, no statistical difference was found between HBOT-2 and HBOT-2.5 (*p*-value = 1.000) (Figure 5A).

### Real-time PCR

**Figure 6:**
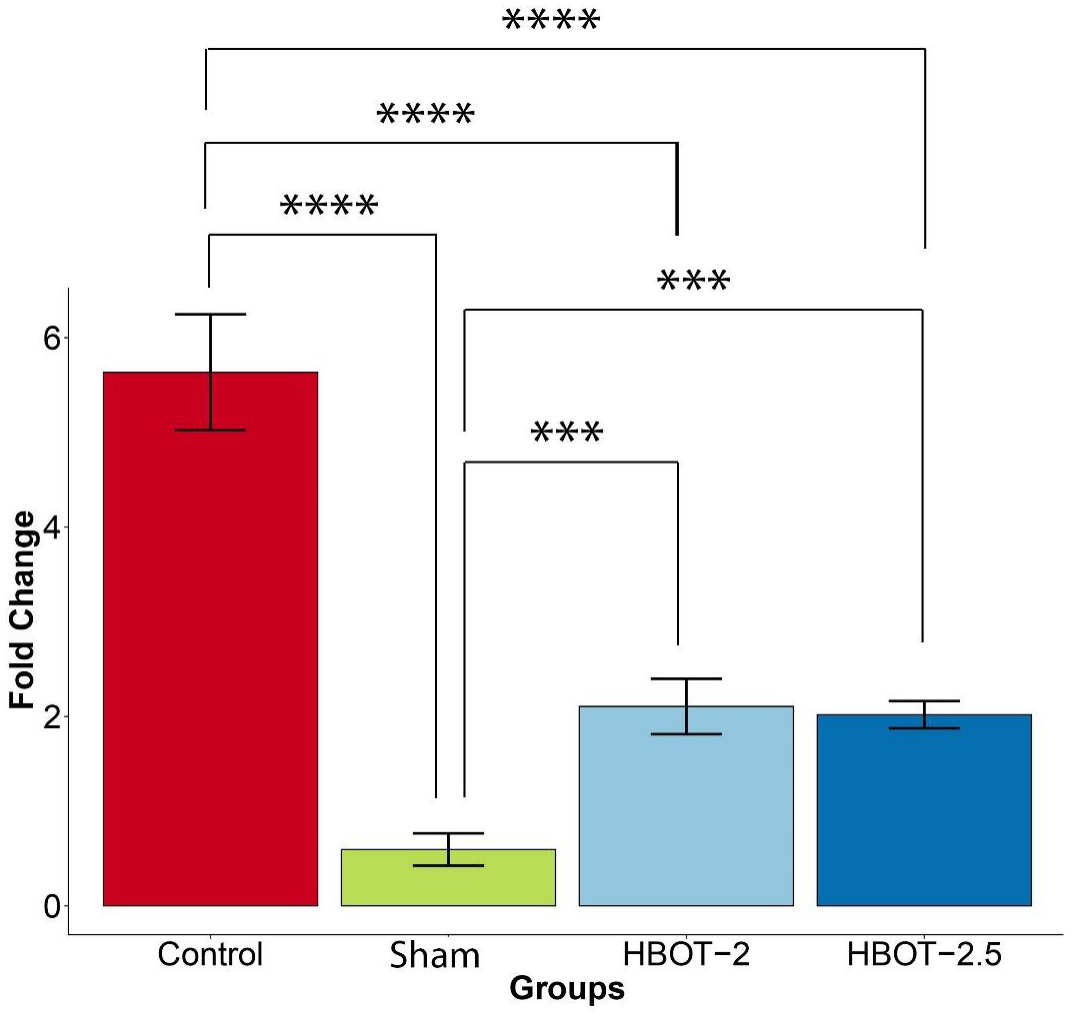
The effects of HBOT on the *GRIN2B* expression. The sham group exhibited significantly lower gene expression compared to the control group (*p*-value ~0). However, both HBOT-receiving groups displayed a notable increase in gene expression in comparison to the sham group (HBOT-2 *p*-value = 0.0062312, HBOT-2.5 *p*-value = 0.0088335). Despite this increase, gene expression in the HBOT-2 and HBOT-2.5 groups remained significantly lower than in the control group (*p*-value~0 for both) (****p-value ≤ 0.0001, ***p-value ≤ 0.001). *n*(Control) = 9, *n*(sham) = 12, *n*(HBOT-2) = 11, *n*(HBOT-2.5) = 12.

Gene expression analysis was conducted in 58-day-old rats. The gene fold data revealed significant differences between groups (*p*-value = 1.82e-12). Tukey’s *post hoc* analysis illustrated that the expression of the *GRIN2B* gene was markedly higher in control rats compared to the sham rats (*p*-value ~0) (Figure 6).

It has been demonstrated in Figure 6 that the *GRIN2B* gene expression has significantly increased in rats after undergoing HBOT [sham and HBOT-2 (*p*-value = 0.0062312), sham and HBOT-2.5 (*p*-value = 0.0088335)].

Despite this observed increase in gene expression post-treatment, the *GRIN2B* expression in both HBOT groups remained notably lower than in the control group [control and HBOT-2 (*p*-value ~0), control and HBOT-2.5 (*p*-value ~0)] (Figure 6).

Furthermore, following the administration of hyperbaric oxygen, there was no significant difference in gene expression between the HBOT-2 and HBOT-2.5 groups (*p*-value = 0.9970551) (Figure 6).

## Discussion

Hyperbaric oxygen therapy shows promise as a potential approach for addressing cognitive disorders, as suggested by emerging research. The first aim of the present study was to investigate the effect of HBOT on several autism-related behaviors and then to measure if this treatment has effects at the gene level in animal models of autism. For the second purpose, we chose the *GRIN2B* gene that plays a role in Glutamate signaling. Our findings confirm that a single injection of VPA on gestational day 12.5 induced autism-like behavioral patterns in rat offspring.

### Morphological abnormalities

Our initial finding revealed that rats exposed to prenatal VPA exhibited tail malformations in 80% of the rats consistent with previous reports (Foley et al., 2012; Kim et al., 2013). In contrast, two studies, one utilizing 500 mg/kg of VPA and the other 600 mg/kg, reported tail malformations in 10 and 34% of rats, respectively (Favre et al., 2013; Saft et al., 2014). These studies suggest that exposure to valproic acid during critical stages of pregnancy, such as neural tube closure, may trigger teratogenic effects in offspring, however, the exact mechanism requires further investigation. Despite these varying observations, understanding the higher incidence of this anomaly in our rats compared to previous studies requires additional research into genetic and embryonic factors.

### Behavioral tests

#### Social interactions

Our findings align with prior research (Bambini-Junior et al., 2011; Jaramillo et al., 2014), indicating that prenatal VPA exposure leads to social deficits in rats. Specifically, VPA-exposed rats tend to spend more time interacting with inanimate objects and familiar individuals, rather than engaging with unfamiliar ones. The results indicated that HBOT at the pressure of 2 and 2.5 ATA generally reduced social reluctance and increased social interaction, consistent with the observations of Luo et al. (2020). In fact, our observations suggest a potential decrease in social anxiety and fear of encountering new individuals in VPA-exposed rats following HBOT.

It’s noteworthy that Spencer et al. (2005) measured the number of active approaches to the stimulus instead of the interaction duration. According to their theory, after the approach has taken place, the duration of the encounter is dependent on both rats, the one who initiates it and the recipient one. Although we did not measure this criterion, we acknowledge its potential significance as we noticed instances where the tested animal attempted interaction, but the stimulus appeared occupied with its own activities, such as grooming or exploring, and paid minimal attention to the tested animal’s presence.

#### Anxiety

This study assessed anxiety levels in rats using the elevated plus maze (EPM), a behavior often linked to autism. According to our results, VPA-exposed rats spent more time in open arms, indicative of lower anxiety levels compared to the control group. While our findings align with some studies (Schneider et al., 2005; Fereshetyan et al., 2021), conflicting research suggests that prenatal VPA exposure actually increases anxiety levels (Jaramillo et al., 2014; Rajizadeh et al., 2019).

Our study’s findings suggest a potential increase in anxiety levels in HBOT-treated rats, contrasting with previous studies that have shown promising outcomes in reducing anxiety levels in VPA-exposed rats (Peng et al., 2010) or even studies indicating no effect of HBOT on anxiety (Fischer et al., 2022). The differences observed in outcomes may be attributed to variations in experimental conditions. Moreover, autistic behaviors vary widely and can appear paradoxical in therapeutic settings due to their complexity and variety. According to a 2022 study (Fischer et al., 2022), there’s a proposed critical age window for HBOT administration to reduce anxiety-like behavior, which might contribute to our observed differences.

#### Exploratory and repetitive behavior

In this study, we employed the Y-maze to measure exploratory behavior, that is, the desire to discover new environments compared to familiar environments, as well as to measure repetitive behavior. Our findings revealed that VPA-exposed rats traveled shorter distances within the maze compared to the control group. This observation might suggest either reduced interest in exploring new environments or heightened anxiety in unfamiliar settings. Post-treatment, the group that received HBOT at 2 ATA displayed nearly the same behavior as the control group, exhibiting a substantial increase in distance traveled. However, in the HBOT-2.5 group, there was a slight decrease in distance covered. This contradictory effect between the two pressures might be linked to increased anxiety levels noted earlier in the HBOT-2.5 group, warranting more detailed investigations into underlying mechanisms.

Interestingly, the percentage of spontaneous alternation behavior in VPA-exposed rats did not significantly differ from the control group, suggesting a lack of pronounced repetitive behavior. This outcome contrasts with findings from some studies (Schneider and Przewłocki, 2004; Pelsőczi et al., 2019; McKinnell et al., 2021) reporting increased repetitive behaviors in similar models. Our findings suggested the potential for HBOT to ameliorate repetitive behavior in VPA-exposed rats; however, as there is a lack of research on HBOT’s effects specifically on rat behavior, we lack comparative data for our results.

Overall, the association between prenatal VPA exposure and repetitive behavior in rats is likely to be complex, multifactorial, and likely influenced by a spectrum of individual and pre/post-natal environmental factors. Further research is crucial to unravel the intricacies of this relationship.

#### Spatial learning and memory

Our study did not reveal any statistical disparities between VPA-exposed rats and the control group in terms of spatial learning and memory, aligning with the findings of certain researchers (Markram et al., 2008; Bambini-Junior et al., 2011). However, contrasting studies have reported impaired spatial learning and memory in VPA-exposed rats (Hou et al., 2018; Rajizadeh et al., 2019; Kim et al., 2022).

In Figure 5, it’s evident that HBOT significantly reduced the escape latency to locate the platform across the five days of the experiment. Similar observations in other studies (Liu et al., 2013; Gottfried et al., 2021), support the notion that HBOT holds the potential for enhancing spatial learning abilities in the VPA-exposed rat model of autism.

#### Molecular studies

Our study revealed significantly reduced expression of the *GRIN2B* gene, responsible for encoding the GluN2B subunit of the NMDA receptor, in VPA-exposed rats compared to the control group. These findings have been consistent across various research, including studies involving autistic patients (Burnashev and Szepetowski, 2015; Hu et al., 2016; Bell et al., 2018). However, contradictory reports exist, showing increased GRIN2B expression in autism (Rinaldi et al., 2007; Chuang et al., 2014), contrasting our results. We believe that the existing contradiction may be due to the complexity of the autism disorder or down-up regulation mechanisms.

According to the findings of our study, HBOT with absolute pressures of 2 and 2.5 atmosphere significantly increased the expression of the *GRIN2B* gene compared to the VPA-exposed group. Yet, even after HBOT, gene expression levels remained lower than those in the control group. Interestingly, there was no variance in gene expression between the HBOT-2 and HBOT-2.5 groups, suggesting that the increase of 0.5 ATA did not elicit a noticeable impact on gene expression.

##### HBOT’ mechanism of action

HBOT can affect behavior in several ways. First, HBOT can increase cerebral blood flow (Chen, 2007). This enhanced oxygen delivery may improve the function of brain cells, including neurons in the PFC, and potentially contribute to improved behaviors. Increased oxygen levels could promote synaptic plasticity and neurotransmission. Also, there are studies that found benefits related to a reduction in cerebral edema, apoptosis, cerebral glucose utilization, vascular density, and synaptic remodeling, each of which has effects on improved functional and cognitive outcomes (Schimmel et al., 2023).

The other impact of HBOT is through inflammation and oxidative stress. Hyperoxia generated during HBOT may stimulate the preservation of IκBα and thereby inhibit NF-κB release, resulting in less gene transcription of pro-inflammatory cytokines and, thus, an anti-inflammatory state despite oxidative stress (De Wolde et al., 2021). Some studies suggest that autism is associated with inflammation and oxidative stress in the brain (Rose et al., 2012; Liu et al., 2022; Usui et al., 2023). HBOT has anti-inflammatory and antioxidant effects. It may help reduce neuroinflammation and oxidative stress, which can contribute to improved gene expression and behavior.

It has been shown that HBOT increases the rate of angiogenesis (Tal et al., 2017; Buckley and Cooper, 2022). This can improve perfusion and balance inflammation which leads to improved autism-related behaviors in VPA-exposed rats. In addition, HBOT has been proposed to enhance neuroplasticity (Boussi-Gross et al., 2013; Efrati et al., 2013). This increased neuroplasticity might play a role in improving social and behavioral deficits in the rat model.

Additionally, HBOT has the capacity to alter gene expression through two primary mechanisms: oxygen-responsive genes and epigenetic modifications. Studies have shown that HBOT can increase the levels of oxygen radicals (De Wolde et al., 2021) although reactive compounds containing oxygen are usually harmful to cells when accumulated to relatively high concentrations, they are also instrumental in the control of the activity of a myriad of proteins, and control both the upregulation and downregulation of gene expression (Hancock, 2021). ROS species can mediate transcription factors, such as hypoxia-inducible factor (HIF), AP-1, ATF, and NF-kappaB (Turpaev, 2002; Hancock, 2021). So, changes in oxygen levels can lead to alterations in gene expression.

Epigenetic changes, such as DNA methylation and histone modifications, play a crucial role in regulating gene expression. Some research suggests that HBOT has been associated with changes in DNA methylation patterns and, hence it may potentially lead to changes in gene expression profiles (Liu et al., 2020).

Given the crucial function of this gene in the modulation of glutamatergic signaling and in the regulation of synaptic activity, its aberrant activation has been implicated in several neurological disorders, including autism (Yoo et al., 2012; Sceniak et al., 2019). Based on some research, a decrease in the expression or function of the *GRIN2B* gene can impair NMDA-dependent signaling and can disrupt the excitatory-inhibitory balance of the middle area of the frontal lobe causing some autism-like behaviors such as social interaction disorders (Rudebeck et al., 2008; Yoo et al., 2012; Lee et al., 2016; Bell et al., 2018). The increased expression of the *GRIN2B* gene due to HBOT suggests a potential link to the improvement observed in behaviors. This elevation in *GRIN2B* expression might influence NMDA receptor function, which could potentially impact the excitation-inhibition balance in the brain.

## Conclusion

Generally, the group that got HBOT at a pressure of 2 ATA showed greater improvement in social interaction and exploratory behaviors, and the group that received HBOT at a pressure of 2.5 ATA exhibited better improvement in the social novelty test. Molecular studies revealed the downregulation of the *GRIN2B* gene in VPA-exposed rats. HBOT at varying pressures enhanced *GRIN2B* gene expression, suggesting a potential mechanism for behavioral improvement.

Finally, these findings strongly suggest that HBOT holds promise as a potential intervention for addressing the impairments associated with autism. However, further investigation is warranted to thoroughly understand its mechanisms and optimize its application for clinical intervention.

## Data availability statement

The original contributions presented in the study are included in the article/supplementary materials, further inquiries can be directed to the corresponding author.

## Ethics statement

The animal study was approved by Iran National Committee for Ethics in Biomedical Research. The study was conducted in accordance with the local legislation and institutional requirements.

## Author contributions

YM: Investigation, Methodology, Writing – original draft. AM: Conceptualization, Resources, Supervision, Writing – review & editing. MK: Conceptualization, Writing – review & editing. MF: Methodology, Supervision, Writing – review & editing. DH: Investigation, Writing – original draft. JT: Methodology, Writing – review & editing.
